# Supplementary material for: Empirical methods for controlling false positives and estimating confidence in ChIP-Seq peaks
Source: BMC Bioinformatics. 2008 Dec 5;9:523. doi: 10.1186/1471-2105-9-523 (PMC2628906; doi:10.1186/1471-2105-9-523)
Supplement: Additional File 1 — A variety of html documents from the USeq web site detailing the available applications, their best usage, output file type descriptions, command line menus, etc. [file 1471-2105-9-523-S1.zip › USeqUserGuides/JavaDoc.zip!/JavaDoc/trans/cel/ChromosomeIndex.html]

ChromosomeIndex


|  |  |  |
| --- | --- | --- |
| **Overview** \| **Package** \| **Class** \| **Use** \| **Tree** \| **Deprecated** \| **Index** \| **Help** | |  |
| **PREV CLASS**   **NEXT CLASS** | **FRAMES**    **NO FRAMES**     **All Classes** |  |
| SUMMARY: NESTED \| FIELD \| CONSTR \| METHOD | DETAIL: FIELD \| CONSTR \| METHOD |  |


---


## trans.cel Class ChromosomeIndex

```
java.lang.Object
  trans.cel.ChromosomeIndex
```

---

``` public class ChromosomeIndex extends java.lang.Object ```

Use to keep track of intensities in a array, their chromosome name, and start stop indexes.

---

**Constructor Summary**

`ChromosomeIndex(java.lang.String chromosome,
int startIndex,
int stopIndex)`


| **Method Summary** | |
| --- | --- |
| `java.lang.String` | `getChromosome()` |
| `int` | `getStartIndex()` |
| `int` | `getStopIndex()` |

**Methods inherited from class java.lang.Object**

`equals, getClass, hashCode, notify, notifyAll, toString, wait, wait, wait`

**Constructor Detail**

### ChromosomeIndex

```
public ChromosomeIndex(java.lang.String chromosome,
                       int startIndex,
                       int stopIndex)
```


**Method Detail**

### getChromosome

```
public java.lang.String getChromosome()
```

---


### getStartIndex

```
public int getStartIndex()
```

---


### getStopIndex

```
public int getStopIndex()
```


---


|  |  |  |
| --- | --- | --- |
| **Overview** \| **Package** \| **Class** \| **Use** \| **Tree** \| **Deprecated** \| **Index** \| **Help** | |  |
| **PREV CLASS**   **NEXT CLASS** | **FRAMES**    **NO FRAMES**     **All Classes** |  |
| SUMMARY: NESTED \| FIELD \| CONSTR \| METHOD | DETAIL: FIELD \| CONSTR \| METHOD |  |


---
